# Rapid bone staining with hair removal (RAP-B/HR): a non-destructive and rapid whole-mount bone staining protocol optimized for adult hairy mice

**DOI:** 10.1038/s41598-021-81616-7

**Published:** 2021-01-21

**Authors:** Nobuo Kariyama, Hiromi Sakata-Haga, Tsuyoshi Tsukada, Hiroki Shimada, Makoto Taniguchi, Toshihisa Hatta

**Affiliations:** 1 Department of Anatomy, Kanazawa Medical University, Ishikawa, Japan; 2 Department of Physical Therapy, Kanazawa Rehabilitation Academy, Ishikawa, Japan; 3 Department of Neurosurgery, Kanazawa Medical University, Ishikawa, Japan; 4 Department of Medical Science, Kanazawa Medical University, Ishikawa, Japan; 5 Department of Life Science, Medical Research Institute, Kanazawa Medical University, Ishikawa, Japan

**Keywords:** Imaging, Musculoskeletal system, Rheumatoid arthritis

## Abstract

We developed a non-destructive and rapid whole-mount bone staining method for small fish, *Xenopus laevis*, and rodent fetuses (RAP-B). RAP-B does not require skin or soft tissue removal. However, RAP-B requires hair removal from hairy animals, such as adult mice and rats. In the present study, we investigated hair removal chemical treatments that did not result in soft tissue destruction. The hair removal effectiveness was investigated using a calcium mercaptoacetate or sodium mercaptoacetate solution on skin fragments obtained from the back of adult mice. A mixture of 2% sodium mercaptoacetate in 3% potassium hydroxide was found to be the most effective in complete hair removal from the skin. Using this hair removal treatment as a pretreatment for RAP-B, the preparation of fast-acting artifact-free whole-mount bone staining was possible without skin and soft tissue removal (RAP-B/HR). We performed a seamless observation from a low magnification wide-view to a high magnification without artifactacting artifacts using fluorescence zoom microscopy. Therefore, the combination of RAP-B/HR and fluorescent zoom microscopy is a novel platform for three-dimensional, wide-field, high-resolution pathological anatomical analysis.

## Introduction

Observing the skeletal system is useful for understanding the normal structure and analyzing various bone pathological changes and/or animal model joint diseases. Anatomical dissection is a traditional approach for examining the skeletal system that facilitates direct observation of the bones and joints, as well as the surrounding tissues including ligaments, tendons, and muscles. In contrast to the anatomical dissection, staining the whole-mounted bone or double staining of the bone and cartilage is a valid alternative approach for studying the skeletal system^1^. Dawson developed a method for staining a whole-body skeletal system with alizarin red S after clearing the specimens^2^. Thereafter, a method using trypsin as a digestive enzyme instead of potassium hydroxide (KOH) for transparency treatment was invented^3^. Whole-mount transparent skeletal preparations immersed in glycerin are useful for observing normal development and studying skeletal malformations in teratology studies. When testing for chemical and drug reproductive toxicity, a detailed examination of the skeletal system is required for fetuses and/or in experimental postnatal pups^4^.

Removal of the epidermis and internal organs is essential for preparing stained bone specimens when performing the tissue clearing method. This process is extremely time-consuming and may cause artifacts due to tissue destruction during preparation. To address these issues, we have developed a new protocol for whole-mount bone staining (Rapid Bone Staining; RAP-B) for small fish and *Xenopus laevis*^5^**,** RAP-B is an innovative and novel method wherein the specimen is simultaneously fixed and cleared using a reagent called RAP-Fix. RAP-Fix is a clearing fixative which contains formalin, a surfactant (Triton X-100), and KOH. RAP-B does not require the removal of soft tissues, including the skin. This non-destructive property of RAP-B provides substantial advantages for studying detailed bone, joint, and surrounding tissue associations. The simple composition and rapid fixing power as well as the high clarity and non-destructive properties of RAP-B render this method superior over conventional methods for preparation of bone-stained specimens. This method obtains specimens with few artifacts, that are capable of high-resolution morphological analysis, such as observing detailed associations between the bone and cartilage. Recently, micro-computed tomography (micro-CT) was used to observe laboratory animal skeletons^6,7^, however, it is not commonly used. Stained bone specimens prepared by RAP-B offer high clarity of the soft tissue, thereby facilitating deep observation using fluorescence or laser confocal microscopy^5^. It may be possible to obtain high-definition deep images with fluorescence microscopy that are comparable to micro-CT images using RAP-B to prepare adult mice and rat whole-body skeletal systems stained with alizarin red S.

Adult mice and rats are used as models of arthritis and other bone-and joint-related diseases. They have body hair and thick soft tissues, such as skin and muscle. These tissues are typically removed when making whole-body transparent skeletal preparations. However, there are concerns regarding the tissue structure and skeleton destruction artifacts due to removal of the hair, skin, and muscle. By contrast, RAP-B provides whole-mount pre- or postnatal pup specimens, that are almost hairless and are transparent without removing the skin or soft tissues. However, for hairy specimens, such as adult mice or rats, specimen hair removal remains a requirement even with RAP-B. In the present study, we examined a non-destructive hair removal treatment without peeling to establish a new pretreatment procedure, which was optimized for RAP-B. We used mercaptoacetic acid [MAA, also known as thioglycolic acid (TGA)], which is widely used as a hair remover, to develop an effective hair removal treatment for hairy animals^8–10^. MAA is an agent that shows acceptable degradation and is reportedly safe and convenient for skin treatments, such as for plantar warts^11^.

In the present study, the hair removal effectiveness using　calcium mercaptoacetate (CMa) or sodium mercaptoacetate (SMa) was evaluated in various contents under different reaction conditions. The hair removal method using CMa and/or SMa was optimized to RAP-B for establishing a modified RAP-B procedure— RAP-B with hair removal treatment (RAP-B/HR)—for hairy animal whole-mount bone staining. To evaluate RAP-B/HR as a tool for non-destructive in situ bone and joint pathological investigation, fluorescence zoom microscopy was performed to delineate the bone and joint lesion in a mouse model of collagen-induced arthritis (CIA).

## Results

### Examining the hair removal conditions

**Figure 1:**
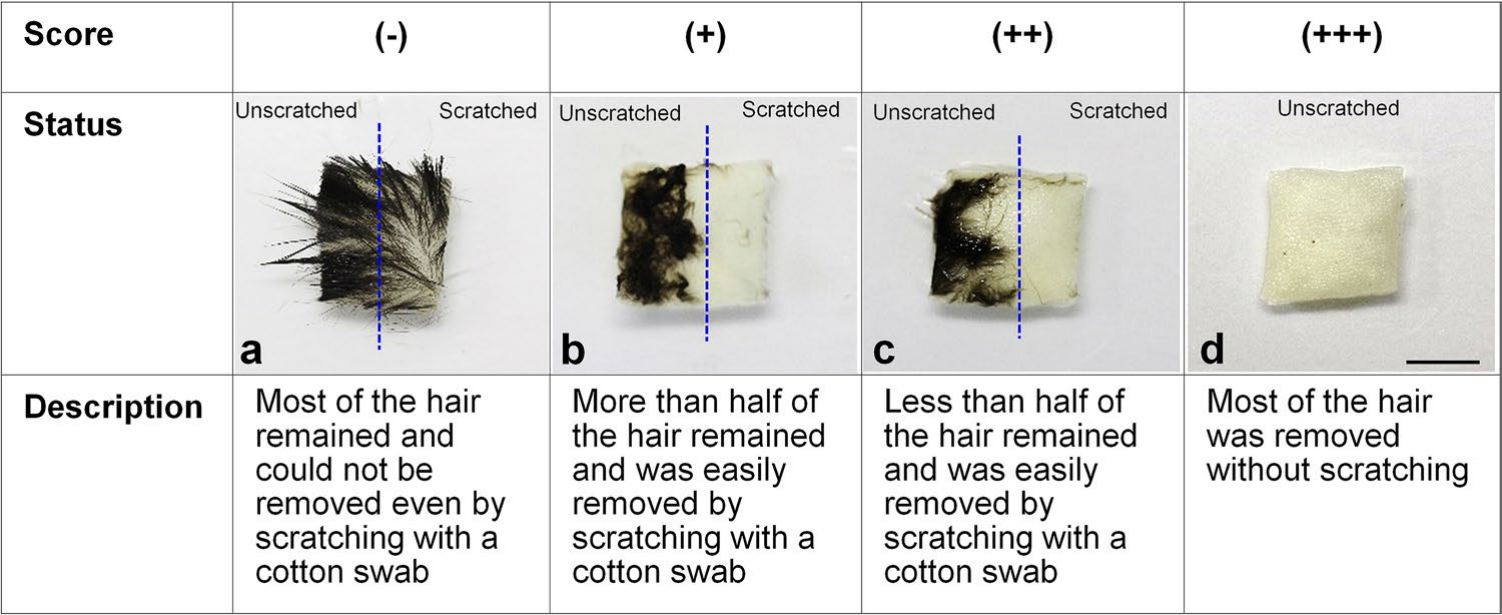
Rating scale system for evaluating the effectiveness of the hair removal treatment effects. Scale bars: 5 mm.

The hair removal treatment effectiveness was evaluated using a scaled rating system (Fig. 1). The hair removal effectiveness by CMa and SMa solution is shown in Table 1. CMa and KOH concentration for hair removal was examined in three compositions − 1% CMa and 1% KOH, 2% CMa and 1% KOH, and 4% CMa and 2% KOH − to determine the most effective concentration. All solutions removed hair without scratching, after 48 h at 42 °C (Table 1). However, CMa did not completely dissolve in distilled water at high concentrations (> 4%), its solubility decreased further, and CMa precipitation was more pronounced when the KOH concentration increased. Therefore, a mixture of 4% CMa and 2% KOH was considered as the upper concentration limit. The same experiment was conducted with SMa, which exhibited a higher solubility in water compared with CMa. The hair removal effectiveness of various SMa and KOH solutions was examined in three compositions − 2% SMa and 1% KOH, 2% SMa and 2% KOH, and 2% SMa and 3% KOH. The solution that provided the fastest and most complete hair removal without scratching was 2% SMa and 3% KOH at 42 °C for 16 h (Table 1). Therefore, we decided to use a mixture of 2% SMa and 3% KOH for 16 h at 42 °C as a hair removal treatment for the following immersion procedure.

**Table 1:** Effects of CMa/KOH and SMa/KOH effects on hair removal.

	(Treatment)				
	12 h	16 h	24 h	48 h	72 h
**CMa/KOH**					
1%/1%	−	−	−	+++	+++
2%/1%	−	−	−	+++	+++
4%/2%	−	−	−	+++	+++
**SMa/KOH**					
2%/1%	+	+	+	++	++
2%/2%	+	+	++	+++	+++
2%/3%	+	+++	+++	+++	+++

*CMa* calcium mercaptoacetate, *SMa* sodium mercaptoacetate.

**Figure 2:**
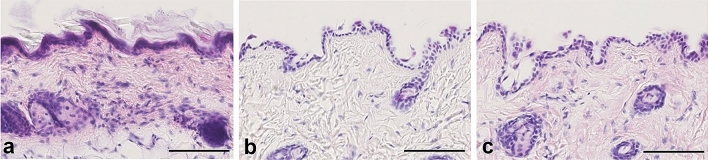
Skin histological changes following hair removal treatment (HE staining). The stratum corneum remained in the control skin (**a**; incubated with distilled water at 42 °C for 16 h). By contrast, the corneum was removed by the hair removal treatment (b and c; incubated in a mixture of 2% sodium mercaptoacetate and 3% KOH at 42 °C for 16 h). However, the hair removal treatment was not affected by the dermal histological structures. The skin after hair removal showed less eosinophilic property throughout the section, particularly in collagen fibers of the dermis (**b**). However, the eosinophilic property was restored by ethanol acetate immersion before staining (**c**). Scale bars: 100 µm.

To confirm the effects of the hair removal treatment on the tissue architecture, we prepared paraffin skin sections following hair removal treatment and examined the hematoxylin and eosin (HE) -stained tissues (Fig. 2). In the skin removed by 2% SMa and 3% KOH (Fig. 2b, c), the stratum corneum disappeared, and the epidermis was thinner compared with that in the control incubated with distilled water (Fig. 2a). However, no marked changes were observed in the dermis. Skin sections following hair removal treatment showed reduced eosin dyeability (Fig. 2b), whereas ethanol acetate immersion (ethanol:acetic acid:distilled water = 95:3:2) before HE staining restored the eosin dyeability (Fig. 2c).

### Staining bone using RAP-B/HR

**Figure 3:**
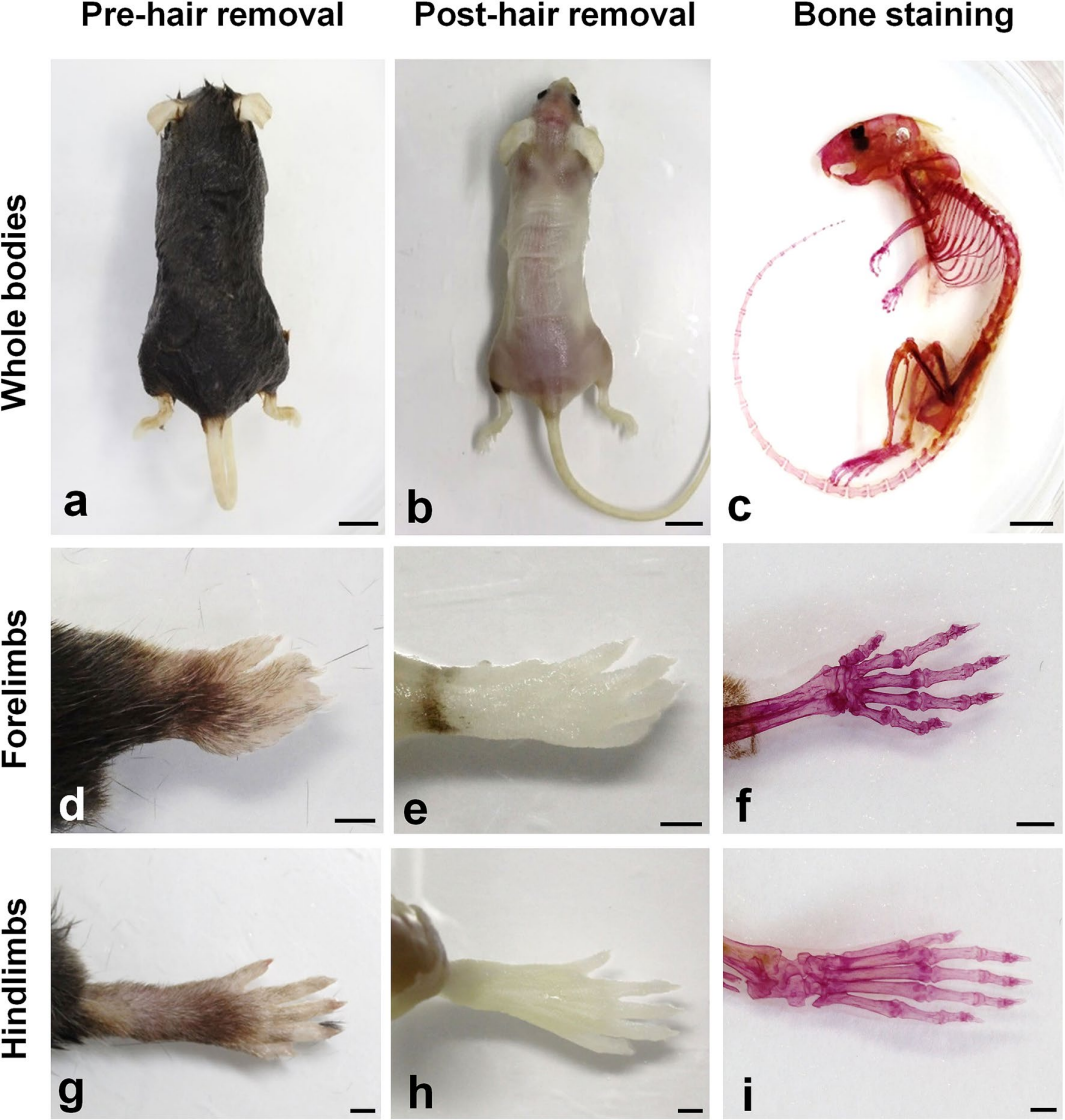
Adult mouse whole-mount bone staining using a modified RAP-B modified with hair removal (RAP-B/HR). Before whole-mount bone staining, RAP-B, specimens were incubated in a mixture of 2% sodium mercaptoacetate and 3% KOH for hair removal. However, the soft tissues, including the skin and muscles, were left behind (**a**, **d**, and **g** vs. **b**, **e**, and **h**, respectively). Subsequently, specimens were stained using the RAP-B procedure and cleared with BABB, then dehydrated with methanol (**c**, **f**, and **i**). Scale bars in **a**–**c**: 1 cm; in **d**–**i**: 2 mm.

Bone staining was performed using RAP-B/HR. As a pretreatment, the viscera were removed after fixing with 4% paraformaldehyde in 50 mM phosphate buffer (pH7.4, PFA). Specimens were decolored using RAP-Fix and RAP-promoting solutions^5^. At this point, the fixing efficacy was not apparent from the external examination because the body hair was still present (Fig. 3a). Subsequently, the hair was removed using 2% SMa and 3% KOH, resulting in whole-mount specimens with semi-transparent skin and muscle (Fig. 3b). Thereafter, the specimens were dehydrated with methanol and immersed in benzyl alcohol and benzyl benzoate (BABB). The soft tissues became completely transparent, and the whole-body skeleton was stained with alizarin red S (Fig. 3c). Moreover, stained mouse limb bone specimens were prepared using RAP-B/HR and examined by macroscopic and light microscopy (Fig. 3d–i). Fine limb body hairs (Fig. 3d, g) were completely removed via the hair removal treatment (Fig. 3e, h). In addition, although the skin was not removed, RAP-B stained the limbs. The soft tissues became transparent and the skeleton was clear in detail (Fig. 3f, i).

### Fluorescence imaging of the CIA mouse model

**Figure 4:**
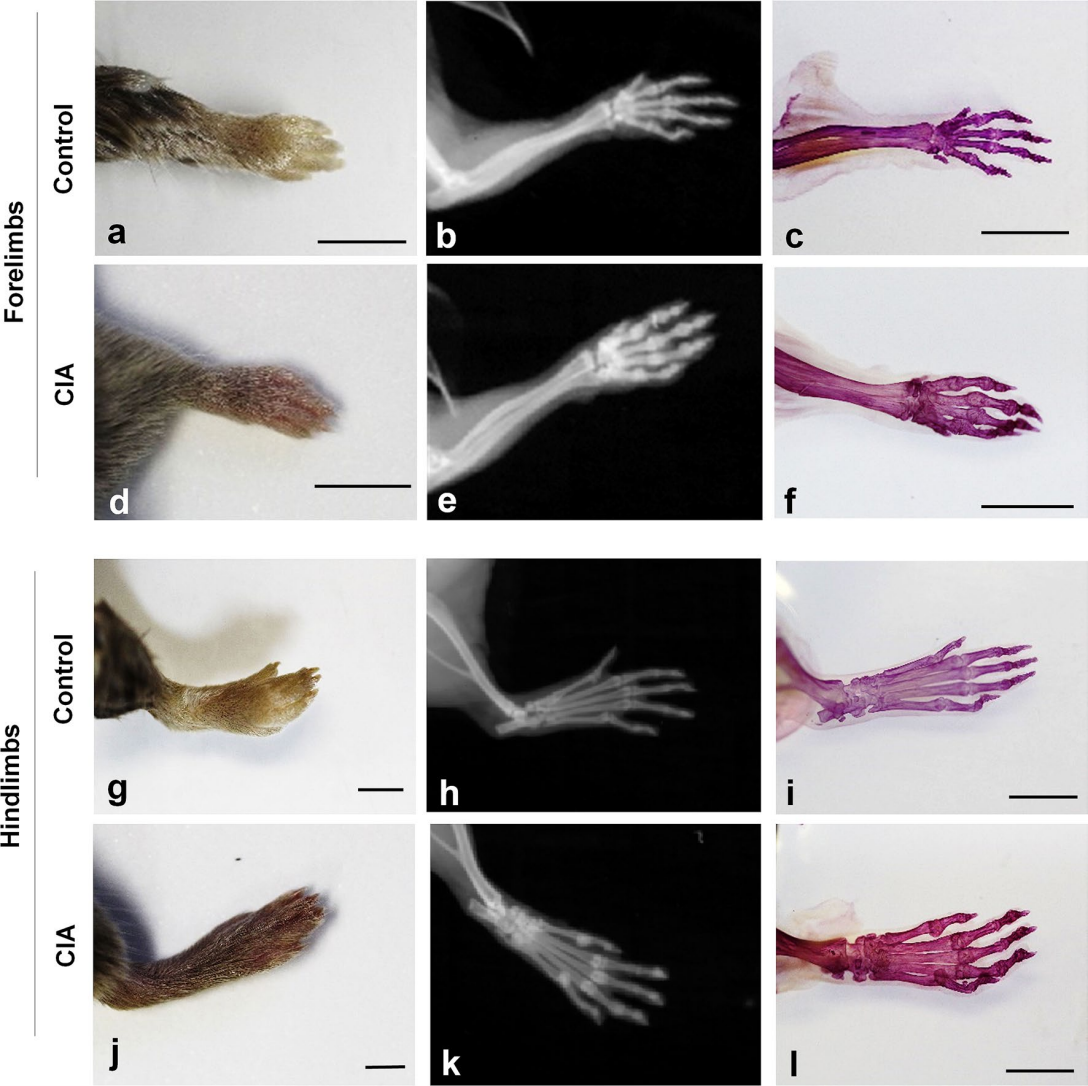
RAP-B modified with hair removal (RAP-B/HR) application examined in CIA mice. The bone and joint pathological changes in CIA mice were not detected by external observation (**a**, **d**, **g**, and **j**). Swelling joints were observed in living mice X-ray images, which were not found in the extremities of a control mouse (**e** and **k** vs. **b** and **h**, respectively). Additional detailed changes in bone and joints could be observed in specimens treated with RAP-B/HR. (**c**, **f**, **i**, and **l**). The arthritic mouse joints were swollen. Scale bars: 5 mm.

**Figure 5:**
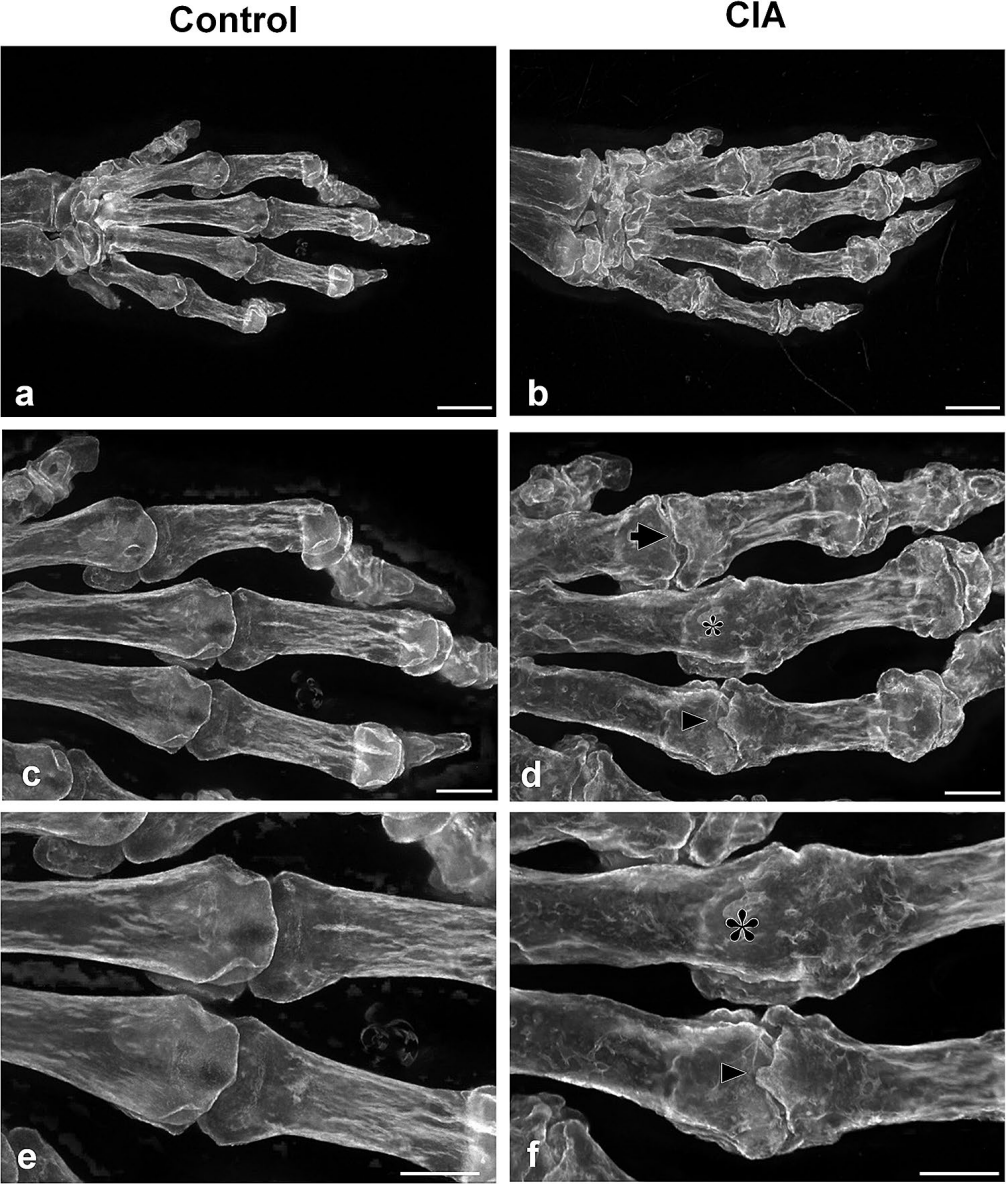
Extended-focus fluorescent images of the phalanges and their joints in the control (**a**, **c**, and **e**) and CIA mice (**b**, **d**, and **f**). Bone erosion was frequently observed in the phalanges of CIA mice. In the metacarpophalangeal joints of CIA mice, the articular surface in both the head of the metacarpals and the base of the proximal phalanx was uneven, but not smooth. In addition, the cavum articulare was narrow (arrows in **d**). Occasionally, the head of the metacarpals and the base of the proximal phalanx were fused (synarthrosis), and the cavum articulare disappeared (asterisks in **d** and **f**). Phalanges osteophytes were observed from the articular surface (arrowhead in **f**). Scale bar in **a**–**b**: 1 mm; in **c**–**f**: 500 µm.

**Figure 6:**
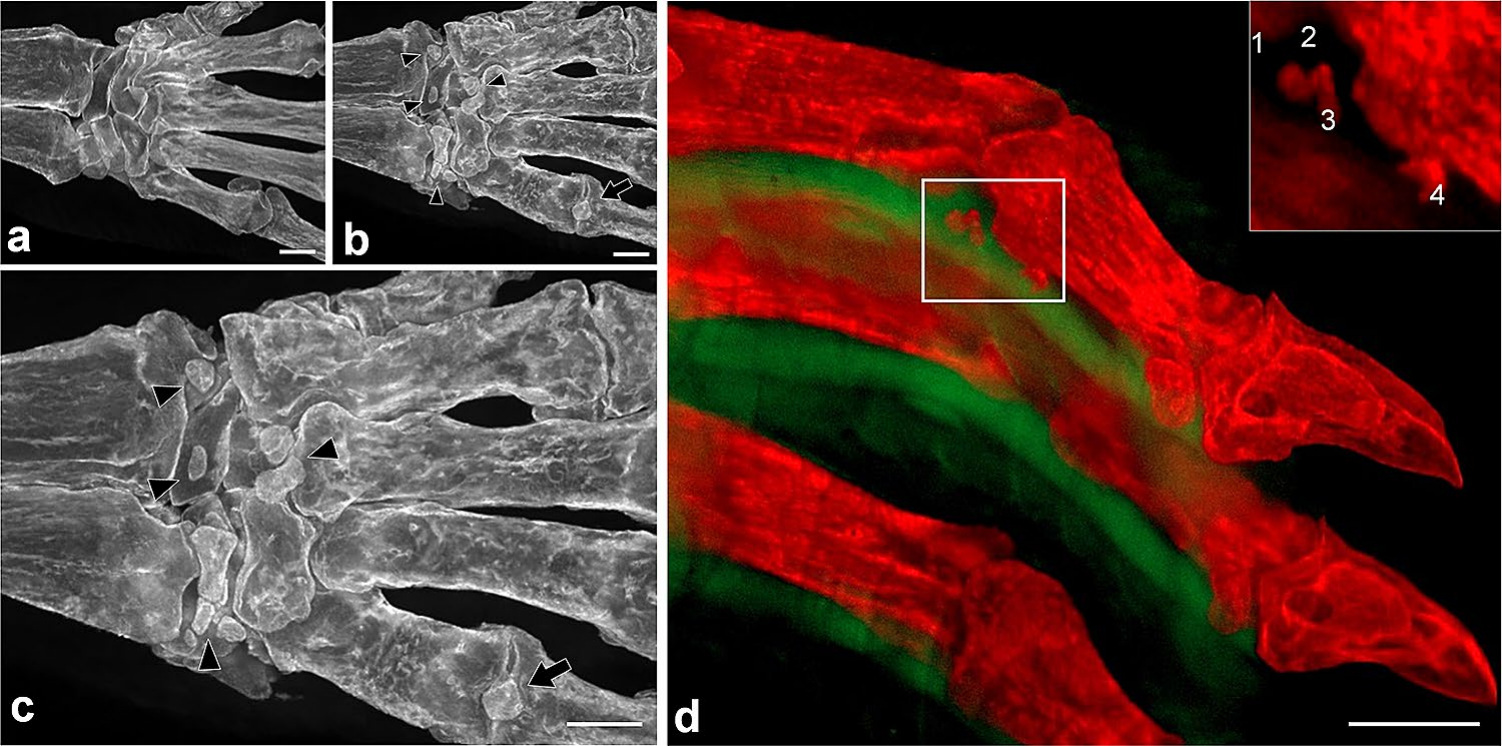
The carpal region fluorescent images (palm-side view) of the control (**a**) and CIA mice (**b**). Several small bones typically not found in the extremities of control mice were observed in the CIA mice (arrowheads in **b** and **c**). The high-power view of **b** is shown in **c**. The heterotopic small bones were also found on the palm side of the 5th metacarpophalangeal joint (arrows). Double fluorescent images of the phalanges (lateral view) in the CIA mice (**d**). An extended-focus image was reconstructed from serially scanned images (35 slices with 100 µm-interval). Red channel (Ex.559–585/Em. 600–690) for alizarin red S and green channel (Ex 450–490/Em. 500–550) for a non-specific fluorescent signal. Multi-microfractures were detected on the insertions of the flexor tendon of phalanges (**d**). Microfractures maximum lengths of (1–4 in an inset of **d**) were measured with ImageJ software (1: 76 µm, 2: 57 mm, 3: 68 µm, 4: 95 µm). Scale bar in d: 500 µm.

We prepared limbs of the CIA mouse model using RAP-B/HR (Fig. 4). Before bone staining, the limb was grossly compared with the control limbs. It was unclear whether bone lesions occurred (Fig. 4d, j). X-ray images obtained using the IVIS imaging system revealed joint-bone enlargement in the forefoot and hindfoot digits but did not detect detailed pathological changes in the joints or bone lesions (Fig. 4e, k). When the RAP-B/HR specimens were observed using bright-field macroscopy, arthritic symptoms, including deformed joint swelling were identified compared with the controls (Fig. 4f, l). Moreover, RAP-B/HR specimens observed using fluorescence zoom microscopy revealed severe pathological processes that resulted in joint deformities, joint fissure narrowing, bone fusion, and fine bone surface pathologies, such as bone erosion and osteophyte formation, in the swollen joint area (Fig. 5b, d, f). In the arthritic carpal region (Fig. 6b, c), multiple small free bones were identified in the CIA mouse model that were not observed in the control mice (Fig. 6a). Moreover, the dorsal part of the metacarpophalangeal joint in the CIA mouse model was also delineated with small bones (Fig. 6b, c), which were not observed in the control mice (Fig. 6a). Furthermore, fluorescence zoom microscopy at Ex488/Em560 nm revealed phalangeal muscle–tendon bundles that were delineated. The flexor tendons were found to contain multiple fine free bones that were localized surrounding the joint (Fig. 6d), ranging from 57 to 95 µm (1: 76 µm, 2: 57 µm, 3: 68 µm, and 4: 95 µm in Fig. 6d). The combination of bone staining with RAP-B/HR and deep imaging with fluorescence zoom microscopy may be adequately effective for identifying pathological changes in bones smaller than 100 µm for easy, non-destructive deep screening of a specimen.

## Discussion

Preparation of whole-mount bone staining requires pretreatment, including the removal of body hair, skin, soft tissues, and internal organs^1,12^. Moreover, large specimen preparation is a time-consuming process^13^. Pretreatment with proteolytic enzymes or strong alkaline solutions (KOH or NaOH) can reduce the processing time as well as achieve a high degree of transparency^14–17^. However, it is often difficult to maintain the original position of the skeleton because the main effect of such pretreatments is destructive to the soft tissues. Therefore, exceedingly delicate adjustments are required to optimize the pretreatment process. Recently, several protocols that render biological specimens transparent have been used for detection of genetically induced fluorescence proteins or fluorescent-labeled antibodies^18–25^. Additionally, the biochemical mechanisms for clearing biological tissues have been reviewed and are well summarized^26,27^. Such modern tissue clearing protocols were developed for deep imaging of small specimens, such as mouse embryos or rat brains^18–25^. However, these protocols were not optimized for examining postnatal or adult skeletal systems, because a considerable amount of time is required to clear the whole specimens without removing soft tissues using these clearing protocols. However, soft tissue removal must be avoided to maintain the original position of the bones. To solve this issue, we developed a non-destructive and ultra-rapid bone staining method, RAP-B^5^—a technique that does not require skinning and facilitates the preparation of whole-mount skeletal specimens within in a short period. A major feature of RAP-B-prepared specimens is that the soft tissues are highly transparent while maintaining the tissue architecture. These unique features facilitate deep observation using fluorescence microscopy and alizarin red S staining. We applied RAP-B—treatment that was optimized for small fish and amphibians—for bone staining in adult mice. One of the major differences among small fish, amphibians, and adult mice is the hair on their bodies. Therefore, hair removal treatment was necessary to apply RAP-B to adult mice. We optimized the hair removal from formalin-fixed specimens using MAA, which is a chemical component in hair removal agents and also in the treatment of warts^11^. From this mixture, we established a modified RAP-B as a one-step hair removal treatment (RAP-B/HR). The advantage of RAP-B/HR is the ability to remove hair without removing skin and other soft tissues. Therefore, skeletal artifacts were rarely observed. Furthermore, because soft tissues were made transparent without damaging the histological architecture, the technique facilitates deep observation at the microscopic level (µm). This set up is perfect for observing small bones, articular cartilage, and the surrounding tissues, such as the synovium, nerves, blood vessels, and muscles.

Micro-CT and magnetic resonance imaging (MRI) are typically used to observe the experimental animal skeletal structure^6,7,28,29^. In recent years, the performance of these instruments has significantly improved, thereby allowing for high-resolution skeletal imaging and quantitative morphological analysis. However, these instruments are expensive and require special equipment and operators, which limit their general use. The RAP-B/HR procedure established in this study has a disadvantage compared with micro-CT and MRI. It cannot observe skeletons in live animals. However, RAP-B/HR can produce extremely transparent whole-mount skeletal specimens for observation with simple equipment and reagents. This technique works seamlessly with a fluorescence zoom microscope using both low or high magnification (Figs. 5, 6). Moreover, RAP-B/HR-treated tissues are extremely transparent, thereby facilitating deep Z-stack imaging. Therefore, observing RAP-B/HR-prepared specimens using fluorescence microscopy enables imaging with a resolution that is equal to or better than that of micro-CT or MRI.

High-resolution three-dimensional (3D) skeletal images comparable to scanning electron microscopy images could be easily reconstructed by creating a multifocal image from Z-stack images using the RAP-B/HR staining technique. In the present study, we demonstrated a two-channel fluorescence observation-delineated tiny 50-µm free bone fragments in the hand joint flexor tendon of a CIA mouse model (Fig. 6). This procedure was extremely useful for analyzing arthritic bone lesions. It would be possible to observe these specimens using a confocal laser microscope to obtain even higher resolution and multi-channel observations. Furthermore, the extremely mild tissue destruction caused by RAP-B/HR fixing suggests the possibility of combining RAP-B/HR with fluorescence multi-immunostaining for various antibodies. In the future, we aim to build a new platform that facilitates in situ mapping of pathological changes in the bone and surrounding tissues. This will include the localization of specific marker molecules to synovial, vascular, and lymphoid tissues, and identification of pro-inflammatory cytokines, inflammatory signaling molecules, and phosphorylation statuses at the macroscopic and microscopic levels.

## Materials and methods

### Experimental animals

Adult female C57BL/6J mice were obtained from Japan SLC (Hamamatsu, aged ≥ 8 weeks, 23.3 g ± 3.2 body weight, n = 9) and were maintained in the room under 12 h/12 h light and dark cycle. Mice were perfused from the left ventricle with 4% PFA (Wako, Osaka) under deep anesthesia via intraperitoneal injection using mixed anesthesia containing medetomidine chloride (0.3 mg/kg), midazolam (4 mg/kg), and butorphanol tartrate (5 mg/kg). Following perfusion fixation, the skin was prepared for hair removal treatment. The internal organs were removed and used for the further bone staining procedure.

### The CIA mouse model

To evaluate the utility of the RAP-B/HR bone staining method, type II collagen-induced arthritic male were purchased (DBA/1JJmsSlc, Japan SLC). Following the protocol for artificially inducing CIA in mice^30,31^, equal amounts of the antigen solution containing bovine type II collagen and Freund's incomplete adjuvant containing *Mycobacterium tuberculosis* H37Ra (Difco Laboratories) were mixed to create a collagen emulsion with a final concentration of 4 mg/ml. For first sensitization, 2 intracutaneous emulsion injections of 0.025 ml were administered at the base of the auricle in 7-week-old male DBA/1JJmsSlc mice (19.4 g ± 0.7 body weight, n = 5). At 3 weeks later for second sensitization, the same dose was administered in the ridge skin as the second sensitization. Mice with swelling in the forelimb and hindlimb were selected by gross external surface observation and were used for subsequent skeletal observation.

### Hair removal treatment

To examine the hair removal treatment, the skin was collected from the backs of C57BL/6J mice following perfusion with 4% PFA. Pieces of skin (approcimately 1 × 1 cm) were immersed in a mixture of KOH and CMa or SMa. The composition of the hair removal solution and the treatment time were empirically determined by a scoring mechanism (Fig. 1). The details of each score were as follows: (−), most of the hair remained after treatment and hair could not be removed when the skin was scratched with a cotton swab (Fig. 1a); (+), most of the hair remained after treatment, but the hair was easily removed by rubbing the skin surface with a cotton swab (Fig. 1b); (++), less than half of the hair was removed during treatment and the hair was easily removed by rubbing with a cotton swab (Fig. 1c); (+++), most of the hair was removed during treatment (Fig. 1d) . Following hair removal, the skin was re-fixed with 4% PFA to prepare 10-µm-thick paraffin sections. The skin sections were stained with HE and observed under a light microscope.

### Bone staining with RAP-B

After fixation with 4% PFA in 50 mM PB, the extremities or whole bodies without the internal organs were immersed in the RAP-B fixative^5^—a mixture of 5% formalin (formalin neutral buffered solution (Wako, Osaka, Japan), 5% polyoxyethylene (10) octylphenyl ether (equivalent to Triton X-100, Wako, Osaka, Japan), and 1% KOH (Wako, Osaka, Japan)—at 42℃ for ≥ 48 h, and were subsequently immersed in the RAP enhancement solution^5^ at 42℃ for ≥ 24 h. To remove the hair, the extremities or whole bodies were incubated in a hair removal solution containing 2% SMa and 3% KOH at 42℃ for 16 h. Following hair removal, the specimens were washed by tap water and underwent bone staining was performed. Bone-stained specimens were dehydrated in a graded methanol series and cleared in a BABB mixture^32,33^.

### Detailed imaging of stained bone specimens

Macroscopic images of the specimens were captured using an EOS 60D digital camera (Canon, Tokyo) equipped with a macro lens (EF-S60 mm F2.8, Canon, Tokyo). The HE-stained skin section histological images were captured with the NanoZoomer slide scanner (Hamamatsu Photonics, Hamamatsu, Japan). Stained bone specimens were observed with a binocular microscope (Stemi DV4, Zeiss, Oberkochen, Germany) and photographed using the EOS 60D digital camera (Canon, Tokyo) equipped with a macro lens (EF-S60 mm F2.8, Canon, Tokyo). An Axio Zoom.V16 microscope equipped with an AxioCam HRm camera (Zeiss, Oberkochen, Germany) was used for fluorescent observation. The stained adult mouse limbs were examined with a fluorescence zoom microscope (Axio Zoom, Carl Zeiss Microscopy, GmbH). Alexa Fluor 594 (Ex. 559–585/Em. 600–690 nm) was used for alizarin red S staining and Alexa Fluor 488 (Ex. 450–490/Em. 500–550 nm) was used to detect the non-specific signals from the muscles and tendons. The 3D images were reconstructed using ZEN software (Carl Zeiss Microscopy, GmbH) from Z-stacked images. A radiogram of the extremities of the CIA mice under anesthesia by isoflurane inhalation was obtained using the IVIS Lumina XRMS Series III (IVIS Lumina XRMS Series III, PerkinElmer, MA) under anesthesia by isoflurane inhalation.

### Ethical approval

All procedures in the present study were approved by the Institutional Animal Care and Use Committee of Kanazawa Medical University (No. 2017-7 and 2020-53). Experiments were performed in accordance with the guidelines for animal experiments based on Japanese guidelines and approval was provided by the Ethics Committee of Kanazawa Medical University. This study was carried out in compliance with the ARRIVE guidelines.

## Supplementary Information

Supplementary Information

## Data Availability

All data generated or analyzed during this study are available from the corresponding author on reasonable request.
