# Supplementary material for: Rapid bone staining with hair removal (RAP-B/HR): a non-destructive and rapid whole-mount bone staining protocol optimized for adult hairy mice
Source: Sci Rep. 2021 Jan 21;11:1950. doi: 10.1038/s41598-021-81616-7 (PMC7820006; doi:10.1038/s41598-021-81616-7)
Supplement: Supplementary file 1 — Supplementary Information [file 41598_2021_81616_MOESM1_ESM.pdf]

<Supplementary Information>

Rapid bone staining with hair removal (RAP-B/HR): a non-destructive and rapid whole-mount bone staining protocol optimized for adult hairy mice

Nobuo Kariyama[1, 2], Hiromi Sakata-Haga[1], Tsuyoshi Tsukada[1, 3], Hiroki Shimada[4], Makoto Taniguchi[5], & Toshihisa Hatta[1]

[1]Department of Anatomy, Kanazawa Medical University, Ishikawa, Japan. [2]Department of Physical Therapy, Kanazawa Rehabilitation Academy, Ishikawa, Japan. [3]Department of Neurosurgery, Kanazawa Medical University, Ishikawa, Japan. [4]Department of Medical Science, Kanazawa Medical University, Ishikawa, Japan. [5]Department of Life Science, Medical Research Institute, Kanazawa Medical University, Ishikawa, Japan.

[*]Correspondence and requests for materials should be addressed to T.H (e-mail: thatta@kanazawa-med.ac.jp)

Figure S1

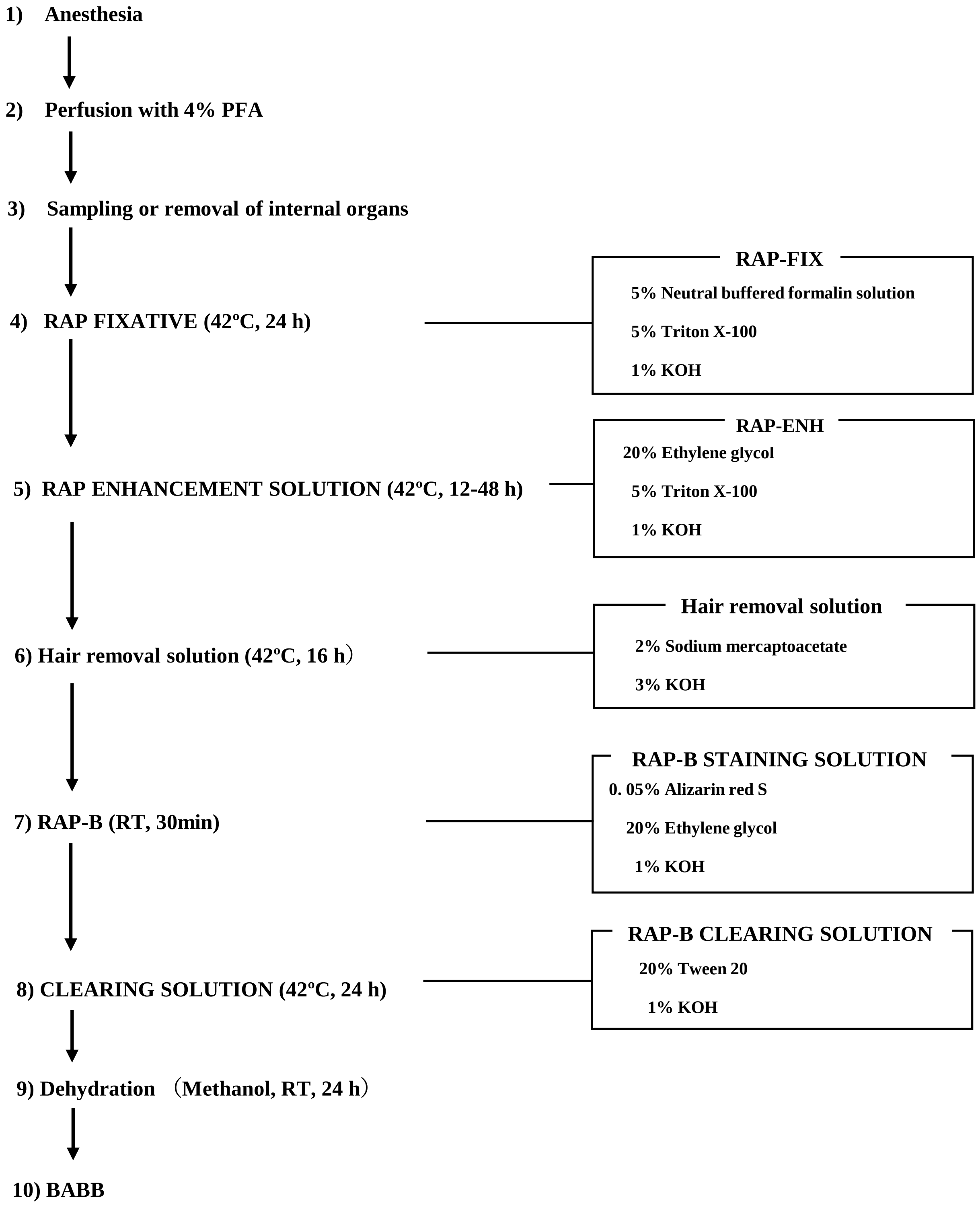

Fig. S1 A new protocol of rapid bone staining for adult mice based on RAP-B. The hair removal procedure was inserted between an immersion to RAP-enhancement solution and immersion to RAP-B staining solution.
